# Tumor Infiltrating PD1-Positive Lymphocytes and the Expression of PD-L1 Predict Poor Prognosis of Soft Tissue Sarcomas

**DOI:** 10.1371/journal.pone.0082870

**Published:** 2013-12-11

**Authors:** Jung Ryul Kim, Young Jae Moon, Keun Sang Kwon, Jun Sang Bae, Sajeev Wagle, Kyoung Min Kim, Ho Sung Park, Ho Lee, Woo Sung Moon, Myoung Ja Chung, Myoung Jae Kang, Kyu Yun Jang

**Affiliations:** 1 Department of Orthopaedic Surgery, Chonbuk National University Medical School, Research Institute of Clinical Medicine and Research Institute for Endocrine Sciences, Jeonju, Republic of Korea; 2 Department of Preventive Medicine, Chonbuk National University Medical School, Research Institute of Clinical Medicine and Research Institute for Endocrine Sciences, Jeonju, Republic of Korea; 3 Department of Pathology, Chonbuk National University Medical School, Research Institute of Clinical Medicine and Research Institute for Endocrine Sciences, Jeonju, Republic of Korea; 4 Department of Forensic Medicine, Chonbuk National University Medical School, Research Institute of Clinical Medicine and Research Institute for Endocrine Sciences, Jeonju, Republic of Korea; University of Texas MD Anderson Cancer Center, United States of America

## Abstract

Recently, the possibility of PD1 pathway-targeted therapy has been extensively studied in various human malignant tumors. However, no previous study has investigated their potential application for soft-tissue sarcomas (STS). In this study, we evaluated the clinical impact of intra-tumoral infiltration of PD1-positive lymphocytes and PD-L1 expression in tumor cells in 105 cases of STS. Intra-tumoral infiltration of PD1-positive lymphocytes and PD-L1 expression were seen in 65% and 58% of STS, respectively. Both PD1-positivity and PD-L1 expression were significantly associated with advanced clinicopathological parameters such as higher clinical stage, presence of distant metastasis, higher histological grade, poor differentiation of tumor, and tumor necrosis. Moreover, both PD1-positivity and PD-L1 positivity were independent prognostic indicators of overall survival (OS) and event-free survival (EFS) of STS by multivariate analysis. In addition, the combined pattern of PD1- and PD-L1-positivity was also an independent prognostic indicator for OS and EFS by multivariate analysis. The patents with a PD1^+^/PD-L1^+^ pattern had the shortest survival time. In conclusion, this study is the first to demonstrate that the infiltration of PD1 positive lymphocytes and PD-L1 expression in STS cells could be used as novel prognostic indicators for STS. Moreover, the evaluation of PD1- and PD-L1-positivity in STS is also available as possible criteria for selection of patients suitable for PD1-based immunotherapy.

## Introduction

Programmed death 1 (PD1) is a member of the CD28 receptor family and attenuates immune responses by negatively regulating T-cell proliferation and function [1,2]. The expression of PD1 on activated T cells, especially on regulatory T cells, enhanced the suppressive function of regulatory T cells [2,3]. The inhibitory effect of PD1 on the activation of T lymphocytes is mediated by the interaction with costimulatory ligands PD-L1 and PD-L2. Especially, the PD1/PD-L1 interaction attenuates the immune response by decreasing cytokine production [4,5] and inducing T lymphocyte anergy and apoptosis [6,7]. The expression rate of PD-L1 in human malignant tumors has been reported to vary from 19% to 92% [8] and the expression of PD-L1 was associated with progression [9-12] and poor prognosis of various human cancers [9,10,13-17]. In addition, intra-tumoral infiltration of PD1-positive T-cells was also positively correlated with the progression of human malignant tumors [11,18-21]. Based on the prognostic impact of the infiltration of PD1-positive lymphocytes and PD-L1 expression in human cancers, PD1 has been put forth as a novel target for immunotherapy of human malignant tumors [8,22-25]. In mouse tumor models, blocking of the PD1 pathway induced tumor regression or prolonged survival of tumor bearing mice [24,25]. Recent reports have shown that anti-PD1 and anti-PD-L1 antibody augmented T-cell proliferation and enhanced humoral immunity [26,27]. In addition, recent preliminary data from clinical trials targeting the PD1-pathway showed response rates of 18 - 31% in human cancers [22,23]. 

Soft-tissue sarcomas (STS) account for less than 1% of human malignant tumors [28] and approximately 50% of STS that were completely resected developed recurrence [29]. Although conventional chemotherapy has shown to benefit patients with advanced STS [30], research into new therapeutic modalities for STS is needed [29]. When considering the important role of the expression of PD1 and PD-L1 in the progression of human malignant tumors, especially for the carcinomas and malignant melanomas, there is reason to believe that the expression of PD1 and PD-L1 could also be involved in STS pathogenesis. Therefore, based on recent evidence of the clinical significance and therapeutic potential of targeting the PD1/PD-L1 interaction in human cancers, we evaluated the clinical impact of the intra-tumoral infiltration of PD1-positive lymphocytes and PD-L1 expression in STS.

## Results

### Association of tumor infiltrating PD1-positive lymphocytes and PD-L1 expression with clinicopathological characteristics of soft-tissue sarcoma patients

**Figure 1:**
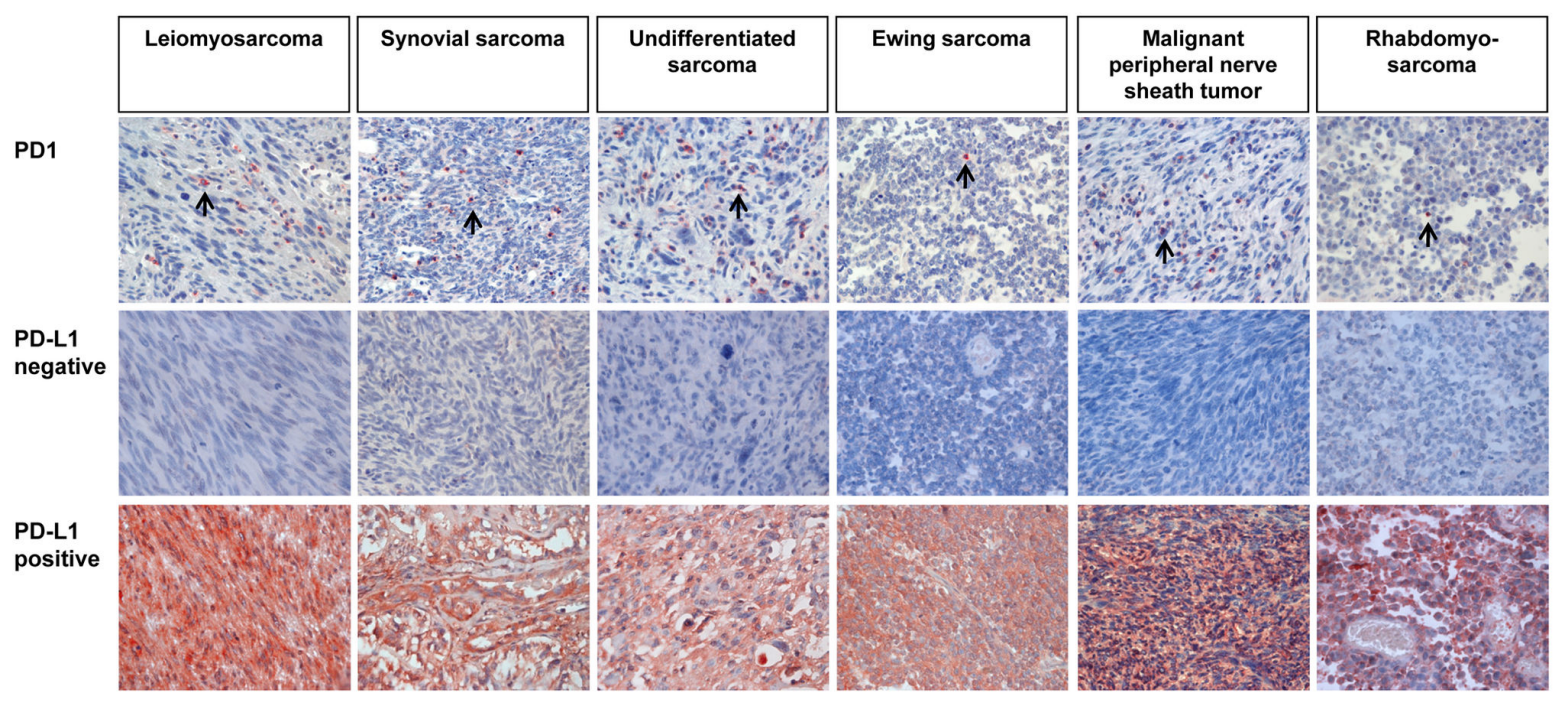
Immunohistochemical expression of PD1 and PD-L1 in various soft-tissue sarcomas. Arrows indicate PD1 positive lymphocytes. Original magnification, x400.

The association of PD1- or PD-L1-positivity with variable clinicopathological factors of STS is summarized in Table 1 and Table 2. PD1 was expressed in tumor infiltrating lymphocytes and PD-L1 was expressed mainly in tumor cells (Figure 1). Intra-tumoral non-neoplastic stromal cells showed negative or weak expression of PD-L1 (Figure S1). Intra-tumoral endothelial cells and inflammatory cells also expressed PD-L1 in some cases (Figure S1). PD-L1 expressing tumor cells escape from the lysis by activated T lymphocytes [31] and the expression of PD-L1 in tumor cells associated with progression of human malignant tumors [9,10,13-17]. Therefore, PD-L1 expression in tumor cells was evaluated by immunohistochemical scoring for PD-L1. Representative negative or positive cases of PD1 or PD-L1 immunostaining in various STS are shown in Figure 1. The mean number of PD1-positive lymphocytes in STS was 18.5 in 10 HPF (mean ± standard error, 18.5 ± 4.6; range, 0-380). The positive subgroups, according to the presence of PD1-positive lymphocytes and PD-L1 expression, were 58% (61 of 105) and 65% (68 of 105), respectively. The presence of tumor-infiltrating PD1-positive lymphocytes was significantly associated with older age of patients, higher tumor stage, distant metastasis, higher histologic, tumor differentiation, mitotic count, and tumor necrosis. PD-L1 expression was also significantly associated with higher tumor stage, deep-seated sarcoma, distant metastasis, higher histologic grade, tumor differentiation, and tumor necrosis. There was possible correlation between PD1-positivity and PD-L1 expression (*P* = 0.063). When we performed additional analysis by combining PD1- and PD-L1-positivity (the PD1/PD-L1 pattern), it was significantly associated with the age of patients, tumor stage, depth of sarcoma, distant metastasis, histologic grade, tumor differentiation, mitotic count, and tumor necrosis (Table 2). When we analyzed tumor stage according to the histologic type of STS, there was tendency towards higher tumor stage for leiomyosarcoma, undifferentiated sarcoma, Ewing sarcoma, angiosarcoma, epithelioid sarcoma, embryonal rhabdomyosarcoma, and clear cell sarcoma (Table S1).

**Table 1:** The expression status of PD1 and PD-L1 according to the histological type of soft-tissue sarcomas.

Histological type	*N*	PD1		PD-L1			PD1/PD-L1	
		positive		positive		-/-	-/+ or +/-	+/+
Leiomyosarcoma	20	9 (45%)		14 (70%)		3 (15%)	11 (55%)	6 (30%)
Synovial sarcoma	16	10 (63%)		12 (75%)		2 (13%)	6 (38%)	8 (50%)
Undifferentiated sarcoma	11	11 (100%)		9 (82%)		0 (0%)	2 (18%)	9 (82%)
Myxoid liposarcoma	10	1 (10%)		3 (30%)		6 (60%)	4 (40%)	0 (0%)
Well differentiated liposarcoma	4	1 (25%)		2 (50%)		1 (25%)	3 (75%)	0 (0%)
Dedifferentiated liposarcoma	3	2 (67%)		2 (67%)		0 (0%)	2 (67%)	1 (33%)
Ewing sarcoma	6	4 (67%)		4 (67%)		0 (0%)	4 (67%)	2 (33%)
Malignant peripheral nerve sheath tumor	6	3 (50%)		3 (50%)		2 (33%)	2 (33%)	2 (33%)
Adult fibrosarcoma	5	3 (60%)		3 (60%)		1 (20%)	2 (40%)	2 (40%)
Angiosarcoma	5	4 (80%)		4 (80%)		1 (20%)	0 (0%)	4 (80%)
Myxofibrosarcoma	4	1 (25%)		1 (25%)		3 (75%)	0 (0%)	1 (25%)
Epithelioid sarcoma	4	4 (100%)		3 (75%)		0 (0%)	1 (25%)	3 (75%)
Alveolar rhabdomyosarcoma	4	3 (75%)		4 (100%)		0 (0%)	1 (25%)	3 (75%)
Embryonal rhabdomyosarcoma	2	1 (50%)		1 (50%)		0 (0%)	2 (100%)	0 (0%)
Pleomorphic rhabdomyosarcoma	2	2 (100%)		2 (100%)		0 (0%)	0 (0%)	2 (100%)
Low grade myofibroblastic sarcoma	2	1 (50%)		0 (0%)		1 (50%)	1 (50%)	0 (0%)
Clear cell sarcoma	1	1 (100%)		1 (100%)		0 (0%)	0 (0%)	1 (100%)

Abbreviations: PD1, programmed death 1; PD-L1, programmed death 1 ligand 1.

**Table 2:** Clinicopathologic variables and the expressional status of PD1 and PD-L1 in soft-tissue sarcomas.

Characteristics		*N*	PD1			PD-L1				PD1/PD-L1		
			positive	*P*		positive	*P*		-/-	-/+ or +/-	+/+	*P*
Sex	female	45	22 (49%)	0.098		27 (60%)	0.376		10 (22%)	21 (47%)	14 (31%)	0.151
	male	60	39 (65%)			41 (68%)			10 (17%)	20 (33%)	30 (50%)	
Age, y	< 60	67	32 (48%)	0.004		40 (60%)	0.149		15 (22%)	32 (48%)	20 (30%)	0.004
	≥ 60	38	29 (76%)			28 (74%)			5 (13%)	9 (24%)	24 (63%)	
Stage	I/II	54	23 (43%)	< 0.001		28 (52%)	0.004		17 (31%)	23 (43%)	14 (26%)	< 0.001
	III/IV	51	38 (75%)			40 (78%)			3 (6%)	18 (35%)	30 (59%)	
Tumor size, cm	≤ 5	33	19 (58%)	0.942		20 (61%)	0.546		8 (24%)	11 (33%)	14 (42%)	0.581
	> 5	72	42 (58%)			48 (67%)			12 (17%)	30 (42%)	30 (42%)	
Depth	superficial	39	18 (46%)	0.057		18 (46%)	0.002		12 (31%)	18 (46%)	9 (23%)	0.005
	deep	66	43 (65%)			50 (76%)			8 (12%)	23 (35%)	35 (53%)	
LN metastasis	absence	90	49 (54%)	0.063		58 (64%)	0.868		19 (21%)	35 (39%)	36 (40%)	0.376
	presence	15	12 (80%)			10 (67%)			1 (7%)	6 (40%)	8 (53%)	
Distant metastasis	absence	74	38 (51%)	0.030		43 (58%)	0.027		19 (26%)	29 (39%)	26 (35%)	0.014
	presence	31	23 (74%)			25 (81%)			1 (3%)	12 (39%)	18 (58%)	
Histological Grade	1	22	5 (23%)	< 0.001		8 (36%)	0.002		11 (50%)	9 (41%)	2 (9%)	< 0.001
	2	35	17 (49%)			22 (63%)			7 (20%)	17 (49%)	11 (31%)	
	3	48	39 (81%)			38 (79%)			2 (4%)	15 (31%)	31 (65%)	
Tumor differentiation	1	9	1 (11%)	< 0.001		6 (67%)	0.012		2 (22%)	7 (78%)	0 (0%)	< 0.001
	2	40	16 (40%)			19 (48%)			16 (40%)	13 (33%)	11 (28%)	
	3	56	44 (79%)			43 (77%)			2 (4%)	21 (38%)	33 (59%)	
Mitotic count	0-9/10 HPF	41	15 (37%)	0.001		22 (54%)	0.162		13 (32%)	19 (46%)	9 (22%)	0.010
	10-19/10 HPF	22	14 (64%)			16 (73%)			3 (14%)	8 (36%)	11 (50%)	
	> 19/10 HPF	42	32 (76%)			30 (71%)			4 (10%)	14 (33%)	24 (57%)	
Tumor necrosis	no necrosis	49	22 (45%)	0.037		24 (49%)	0.007		15 (31%)	22 (45%)	12 (24%)	0.004
	< 50%	42	29 (69%)			33 (79%)			3 (7%)	16 (38%)	23 (55%)	
	≥ 50%	14	10 (71%)			11 (79%)			2 (14%)	3 (21%)	9 (64%)	
Inflammation	minimal	34	17 (50%)	0.355		20 (59%)	0.809		9 (26%)	13 (38%)	12 (35%)	0.818
	mild	47	28 (60%)			31 (66%)			8 (17%)	19 (40%)	20 (43%)	
	moderate	21	13 (62%)			15 (71%)			3 (14%)	8 (38%)	10 (48%)	
	intense	3	3 (100%)			2 (67%)			0 (0%)	1 (33%)	2 (67%)	
PD-L1	negative	37	17 (46%)	0.063								
	positive	68	44 (65%)									

Abbreviations: PD1, programmed death 1; PD-L1, programmed death 1 ligand 1; -/-, PD1-/PD-L1-; -/+, PD1-/PD-L1+; +/-, PD1+/PD-L1-; +/+, PD1+/PD-L1+; LN, lymph node; HPF, high-power fields.

### The infiltration of PD1-positive lymphocytes and the expression of PD-L1 in sarcomas correlate with reduced overall survival and event-free survival according to univariate analysis

**Figure 2:**
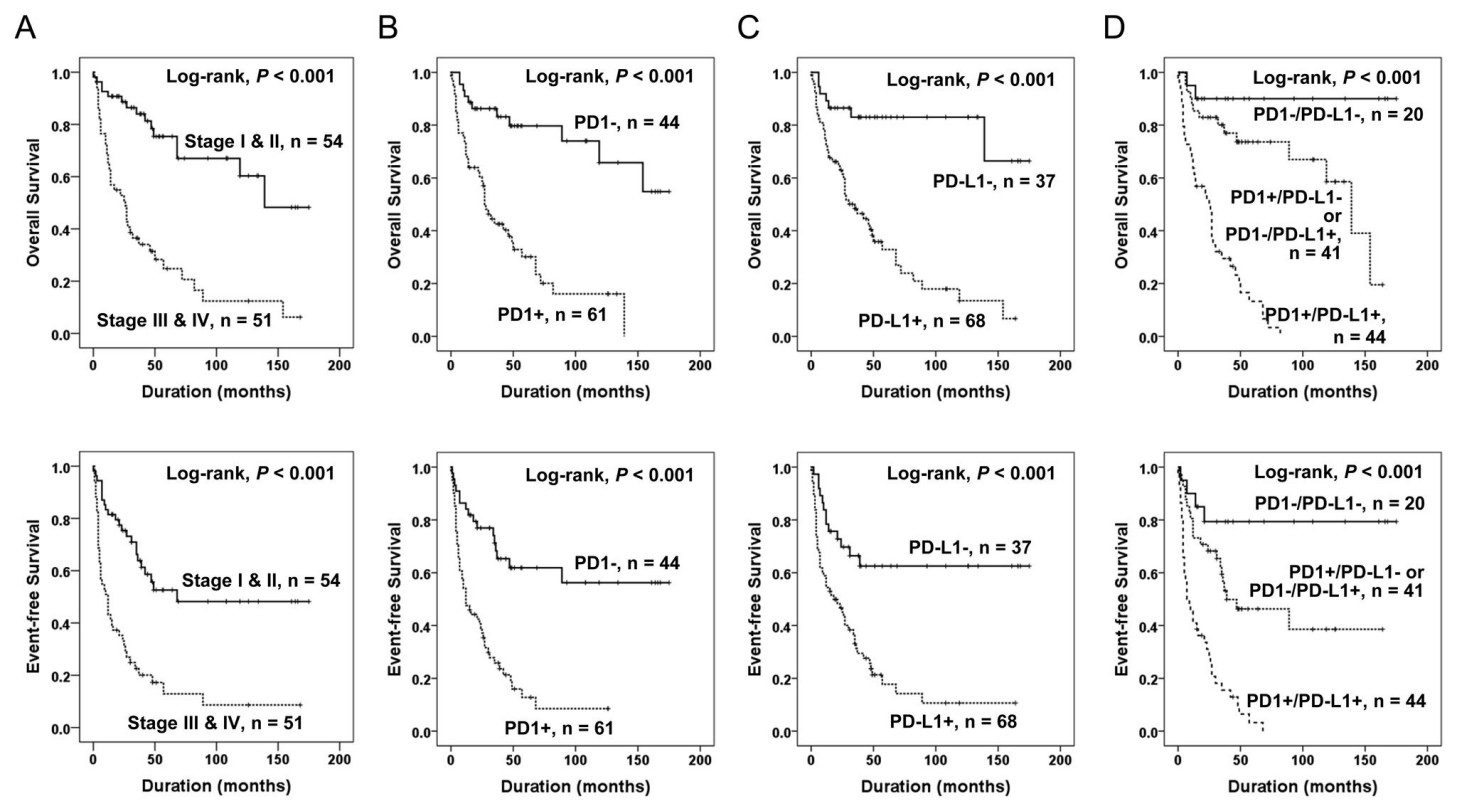
Kaplan-Meier survival analysis in soft-tissue sarcomas. Overall survival and event-free survival according to tumor stage (A), intra-tumoral infiltration of PD1-positive lymphocytes (B), expression of PD-L1 (C), and the combined expression pattern of PD1 and PD-L1 (PD1/PD-L1) (D).

The factors significantly correlated with OS and EFS by univariate analyses were the age of patients, tumor stage, depth of tumor, lymph node metastasis, distant metastasis, histological grade, tumor differentiation, mitotic count, tumor necrosis, intra-tumoral infiltration of PD1-positive lymphocytes, PD-L1 expression, and the PD1/PD-L1 pattern (Figure 2) (Table 3). Intra-tumoral infiltration of PD1-positive lymphocytes predicted shorter OS (*P* < 0.001, HR; 5.068, 95% confidence interval [95% CI]; 2.518-10.201) and EFS (*P* < 0.001, HR; 3.830, 95% CI; 2.157-6.803) (Figure 2 B). The expression of PD-L1 also predicted shorter OS (*P* < 0.001, HR; 5.699, 95% CI; 2.558-12.700) and EFS (*P* < 0.001, HR; 3.274, 95% CI; 1.776-6.036) (Figure 2 C). The PD1/PD-L1 pattern was also significantly correlated with OS (Log-rank, *P* < 0.001) and EFS (Log-rank, *P* < 0.001) (Figure 2 D). The PD1^+^/PD-L1^+^ subgroup showed shorter OS and EFS duration and the PD1^-^/PD-L1^-^ subgroup had the longest OS and EFS. The five-year survival rates of the PD1^-^/PD-L1^-^, (PD1^+^/PD-L1^-^ or PD1^-^/PD-L1^+^), and PD1^+^/PD-L1^+^ groups were 90%, 74%, and 13%, respectively. 

**Table 3:** Univariate Cox regression analysis for overall survival and event-free survival of soft-tissue sarcoma patients.

Characteristics	*N*	OS			EFS	
		HR (95% CI)	*P*		HR (95% CI)	*P*
Sex, male (*vs* female)	60/105	1.457 (0.844-2.516)	0.177		1.245 (0.762-2.034)	0.382
Age, y, ≥ 60 (*vs* < 60)	38/105	2.028 (1.191-3.455)	0.009		2.230 (1.368-3.636)	0.001
Stage, III/IV (*vs* I/II)	51/105	4.504 (2.471-8.210)	< 0.001		3.450 (2.063-5.772)	< 0.001
Tumor size, > 5 cm (*vs* ≤ 5 cm)	72/105	1.561 (0.850-2.867)	0.151		1.161 (0.686-1.965)	0.577
Depth, deep (*vs* superficial)	66/105	3.648 (1.784-7.461)	< 0.001		3.124 (1.728-5.648)	< 0.001
LN metastasis, presence (*vs* absence)	15/105	2.290 (1.204-4.355)	0.011		1.814 (0.968-3.399)	0.063
Distant metastasis, presence (*vs* absence)	31/105	4.065 (2.367-6.980)	< 0.001		4.796 (2.864-8.032)	< 0.001
Histological Grade, 1	22/105	1.000 (Ref)	< 0.001		1.000 (Ref)	0.001
2	35/105	4.478 (1.468-13.665)	0.008		3.196 (1.354-7.545)	0.008
3	48/105	7.822 (2.686-22.777)	< 0.001		4.681 (2.051-10.682)	< 0.001
Tumor differentiation, 1	9/105	1.000 (Ref)	0.009		1.000 (Ref)	0.088
2	40/105	2.011 (0.586-6.895)	0.267		1.818 (0.628-5.267)	0.270
3	56/105	4.171 (1.255-13.857)	0.020		2.704 (0.961-7.609)	0.059
Mitotic count, 0-9/10 HPF	41/105	1.000 (Ref)	0.002		1.000 (Ref)	0.002
10-19/10 HPF	22/105	3.173 (1.447-6.954)	0.004		2.896 (1.472-5.696)	0.002
> 19/10 HPF	42/105	3.403 (1.709-6.775)	< 0.001		2.685 (1.474-4.892)	0.001
Tumor necrosis, no necrosis	49/105	1.000 (Ref)	< 0.001		1.000 (Ref)	0.009
< 50%	42/105	3.009 (1.623-5.580)	< 0.001		2.273 (1.327-3.893)	0.003
≥ 50%	14/105	3.472 (1.557-7.739)	0.002		2.081 (0.991-4.373)	0.053
PD1, positive (*vs* negative)	61/105	5.068 (2.518-10.201)	< 0.001		3.830 (2.157-6.803)	< 0.001
PD-L1, positive (*vs* negative)	68/105	5.699 (2.558-12.700)	< 0.001		3.274 (1.776-6.036)	< 0.001
PD1/PD-L1, -/-	20/105	1.000 (Ref)	< 0.001		1.000 (Ref)	< 0.001
-/+ or +/-	41/105	4.516 (1.004-20.315)	0.049		3.142 (1.073-9.201)	0.037
+/+	44/105	27.706 (6.136-125.102)	< 0.001		10.929 (3.828-31.204)	< 0.001

Abbreviations: OS, overall survival; EFS, event-free survival; HR, hazard ratio; 95% CI, 95% confidence interval; LN, lymph node; HPF, high-power fields; PD1, programmed death 1; PD-L1, programmed death 1 ligand 1; -/-, PD1-/PD-L1-; -/+, PD1-/PD-L1+; +/-, PD1+/PD-L1-; +/+, PD1+/PD-L1+; Ref, reference group.

**Figure 3:**
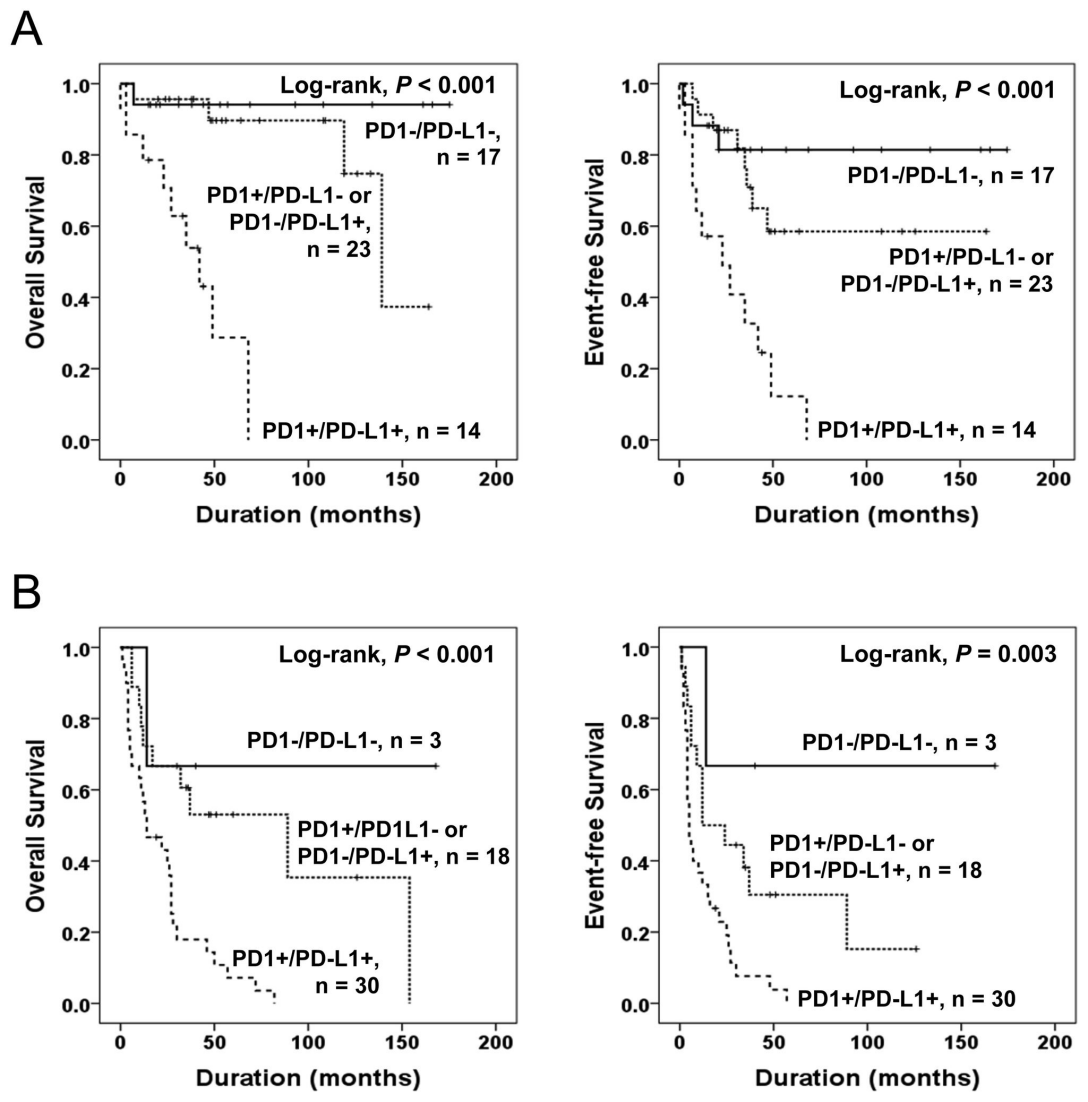
Kaplan-Meier survival analysis in the low and high stage subgroup of soft-tissue sarcomas. Overall survival and event-free survival according to the combined expression pattern of PD1 and PD-L1 (PD1/PD-L1) in low stage (stage I and II) (A) and high stage (stage III and IV) (B) subgroup of soft-tissue sarcomas.

When additional analysis was performed according to the stage of STS patients, the PD1/PD-L1 pattern was also significantly associated with OS and EFS in both low stage (stage I and II) and high stage (stage III and IV) subgroups. In the low stage subgroup, the PD1/PD-L1 pattern was significantly associated with OS (Log-rank, *P* < 0.001) and EFS (Log-rank, *P* < 0.001) (Figure 3 A). In the high stage subgroup, the PD1/PD-L1 pattern was also significantly associated with OS (Log-rank, *P* < 0.001) and EFS (Log-rank, *P* = 0.003) (Figure 3 B). 

The infiltration of PD1-positive lymphocytes, the expression of PD-L1, and the combined expression pattern of PD1 and PD-L1 in soft-tissue sarcomas are independent prognostic factors of shorter event-free survival and poorer overall survival 

The variables included in the multivariate analysis for OS and EFS in Model 1 were age, tumor stage, tumor depth, histological grade, tumor differentiation, mitotic count, tumor necrosis, intra-tumoral infiltration of PD1-positive lymphocytes, and PD-L1 expression. From the multivariate analysis, tumor stage, PD1-positivity, and PD-L1 expression were the independent prognostic indicators which were significantly associated with both OS and EFS. The patients having tumors with an infiltration of PD1-positive lymphocytes had a 4.501-fold (95% CI, 2.132-9.502) greater risk of death (*P* < 0.001) and a 3.174-fold (95% CI, 1.761-5.720) greater risk of shorter EFS (*P* < 0.001). In addition, the patients with PD-L1 expression had a 4.827-fold (95% CI, 2.093-11.131) greater risk of death (*P* < 0.001) and a 2.629-fold (95% CI, 1.407-4.911) greater risk of shorter EFS (*P* = 0.002). When we included the PD1/PD-L1 pattern and excluded PD1- and PD-L1 positivity from the multivariate analysis (Model 2), the PD1/PD-L1 pattern was also an independent prognostic indicator of OS and EFS of STS patients (Table 4).

**Table 4:** Multivariate Cox regression analysis for overall survival and event-free survival of soft-tissue sarcoma patients.

Characteristics	OS			EFS	
	HR (95% CI)	*P*		HR (95% CI)	*P*
Model 1^*^					
Stage, III/IV (*vs* I/II)	2.635 (1.419-4.890)	0.002		2.387 (1.403-4.060)	0.001
PD1, positive (*vs* negative)	4.501 (2.132-9.502)	< 0.001		3.174 (1.761-5.720)	< 0.001
PD-L1, positive (*vs* negative)	4.827 (2.093-11.131)	< 0.001		2.629 (1.407-4.911)	0.002
Model 2^**^					
Stage, III/IV (*vs* I/II)	2.642 (1.422-4.910)	0.002		2.388 (1.403-4.064)	0.001
PD1/PD-L1, -/-	1.000 (Ref)	< 0.001		1.000 (Ref)	< 0.001
-/+ or +/-	3.627 (0.798-16.480)	0.095		2.596 (0.879-7.667)	0.084
+/+	17.898 (3.850-83.208)	< 0.001		7.854 (2.684-22.976)	< 0.001

Abbreviations: OS, overall survival; EFS, event-free survival; HR, hazard ratio; 95% CI, 95% confidence interval; PD1, programmed death 1; PD-L1, programmed death 1 ligand 1; -/-, PD1-/PD-L1-; -/+, PD1-/PD-L1+; +/-, PD1+/PD-L1-; +/+, PD1+/PD-L1+; Ref, reference group. ^*^ The variables included in multivariate analysis for OS and EFS in Model 1 was age, tumor stage, tumor depth, histological grade, tumor differentiation, mitotic count, tumor necrosis, and the expression of PD1 and PD-L1. ** The variables included in multivariate analysis for OS and EFS in Model 2 was age, tumor stage, tumor depth, histological grade, tumor differentiation, mitotic count, tumor necrosis, and the combined expression pattern of PD1 and PD-L1.

## Discussion

In this study, we have shown that 58% of STS had intra-tumoral infiltration of PD1-positive lymphocytes and 65% of STS expressed PD-L1. Both PD1-positivity and PD-L1 positivity were independent prognostic indicators for OS and EFS of STS by multivariate analysis. In addition, the combined PD1/PD-L1 pattern was also an independent prognostic indicator for OS and EFS by multivariate analysis. These findings suggest that the PD1 and/or the PD-L1 expression pattern is very helpful for the prediction of the prognosis of STS patients, and might be used for the selection of patients which could benefit from PD1 pathway-targeted therapy. 

In human malignant tumors, the expansion of PD1-positive lymphocytes was closely correlated with the progression of human malignant tumors. Intra-tumoral infiltration of PD1-positive lymphocytes predicted poor survival in renal cell carcinoma [20,21] and follicular lymphoma [11]. The expansion of PD1-expressing cells was closely correlated with anti-tumor immune modulation, especially when combined with increased intra-tumoral infiltration of FoxP3-positive regulatory T cells [18,21]. Co-infiltration of PD1^+^ cells and regulatory T cells was an independent poor prognostic predictor of renal cell carcinoma [21]. In addition to the prognostic significance of *in situ* infiltration of PD1-positive lymphocytes in tumors, the increase in circulating PD1^+^/CD8^+^ T cells also correlated with the progression and poor outcome of hepatocellular carcinoma patients [19]. Therefore, systemic immunotherapy targeting the PD1 pathway has been proposed as a potentially effective new therapeutic modality for advanced human malignant tumors. Recently, there have been many ongoing trials with PD1 pathway-targeted immunotherapy in various cancers [8]. Recent preliminary data for immunotherapy with an anti-PD1 antibody resulted in response in 27 - 31% of renal cell carcinoma, 28% of melanoma, and 18% of non-small cell lung cancer patients [22,23]. However, there have not been any previous studies investigating the clinical significance of tumor infiltrating PD1-positive lymphocytes to predict the potential of PD1-targeted immunotherapy in STS. In line with previous reports that the infiltration of PD1-positive lymphocytes predicted progression of human malignant tumors, our results are the first to show that the infiltration of PD1-positive lymphocytes was significantly associated with poor prognosis of STS. Therefore, our results strongly suggest that PD1-targeted therapy could also be beneficial to those in the poor prognostic subgroup of STS, which have infiltration of PD1-positive lymphocytes.

In addition to the prognostic significance of intra-tumoral infiltration of PD1-positive lymphocytes, our data also show that the expression of PD-L1 in STS is an independent indicator of poor prognosis of STS patients. In agreement with our results, the expression of PD-L1 has predicted poor survival of breast cancer [9], esophageal cancer [10], pancreatic cancer [13], gastric cancer [14], hepatocellular carcinoma [15], urothelial cancer [16], and renal cell carcinoma patients [17]. Moreover, in addition to the prognostic indicative role of PD-L1 expression in human cancers, the expression of PD-L1 is important for the prediction of the efficacy of PD1-targeted immunotherapy; this is reflected in previous results which demonstrated that 36% of patients having PD-L1^+^ tumors responded to anti-PD1 immunotherapy, while patients with PD-L1^-^ tumors did not [8]. Thus, assessing both PD1-positivity and PD-L1 expression could be powerful tools for the selection of patients which could benefit from PD1-pathway targeted immunotherapy. In our investigation, the co-expression pattern of PD1 and PD-L1 significantly predicted the clinical course of STS. STS with a PD1^+^/PD-L1^+^ phenotype showed the shortest survival time and a more progressive phenotype of STS. Even in low stage STS, the five-year survival rate of the PD1^+^/PD-L1^+^ subgroup was only 29% and the ten-year survival rate was 0%. In contrast, the ten-year survival rates of the PD1^-^/PD-L1^-^ subgroups was 94% in low-stage STS and 67% in high-stage STS. These results suggest that the PD1/PD-L1 interaction has an important role in the progression of STS and might be helpful for the selection of patients which could benefit from PD1-targeted therapy. In this study, STS samples were 65% PD-L1^+^, 58% PD1^+^, and 42% PD1^+^/PD-L1^+^. Therefore, a substantial proportion of STS patients could be a potential candidate for PD1-targeted immunotherapy.

Despite the importance of the PD1/PD-L1 interaction in tumor evasion, the exact mechanism of how the PD1/PD-L1 interaction affects the tumor microenvironment to promote the escape of tumor cells from anti-tumor immunolosurveillance is not clear. Recently suggested mechanisms include that the expression of PD1 in T-cells is increased by tumor-associated antigens or that gamma-chain cytokines are secreted into the tumor microenvironment [15,21]. Subsequently, interferon-γ secreted by PD1^+^/CD8^+^ T cells increase the expression of PD-L1 in tumor cells, which results in tumor evasion via apoptosis induction in cytotoxic CD8^+^ T cells [19].

Recently, there are many ongoing trials with PD1 pathway-targeted therapy of various carcinomas, hematopoietic malignancies, and melanoma. However, there have been no previous studies investigating the potential of such therapies for the treatment of STS. STS is one of the malignant tumors which commonly has poor outcomes and only limited therapeutic options. In this study, we have demonstrated for the first time that the infiltration of PD1 positive cells and PD-L1 expression in STS cells could be used as novel prognostic indicators of STS. Moreover, in addition to the prognostic impact of PD1-positivity for STS, our findings also suggest that the evaluation of PD1- and PD-L1-positivity in STS might be used for the selection of patients suitable for PD1-based immunotherapy. 

## Materials and Methods

### Ethics

This study obtained institutional review board approval from Chonbuk National University Hospital. Written informed consent was provided according to the Declaration of Helsinki.

### Patients and samples

One hundred and forty-five cases of STS patients, other than those with gastrointestinal stromal tumors and Kaposi’s sarcomas, who underwent surgical resection for the primary lesion between July 1998 and December 2011 at Chonbuk National University were included in the present study. However, original histologic slides, paraffin-embedded tissue blocks, or clinical information were not available in thirty-three cases. Thereafter, the remaining 112 cases of STS were reviewed and classified according to the 2013 World Health Organization classification of tumors of soft tissue and bone [28]. Seven cases originally diagnosed as well differentiated liposarcoma were reclassified as atypical lipomatous tumor and excluded from this study. As a result, 105 cases of STS were available for this study (Table 1). Forty-one patients received adjuvant chemotherapy; thirty-four patients received radiation therapy; sixteen patients received both adjuvant chemotherapy and radiation therapy; and forty-six patients received no adjuvant treatment. The median follow-up duration was 35 months (range, 1 - 175). The median survival was 59.9 months and the five- and ten-year survival rates for the entire STS patients were 50% and 36%, respectively. Tumors were graded according to the FNCLCC (French Fédération Nationale des Centres de Lutte Contre le Cancer) system [28], and staged based on the guidelines of the American Joint Committee on Cancer [32]. Clinical information was obtained by reviewing medical records.

### Establishment of tissue microarray and immunohistochemical staining

The tissue microarray (TMA) established after review of original H&E slides. Two 3.0 mm cores were taken from the most intact solid area of the highest histological grade which did not showing necrosis or degenerative change. Immunohistochemical staining for PD1 (1:50, Abcam, clone NAT, Cambridge, UK) and PD-L1 (1:100, Santa Cruz Biotechnology, clone H-130, CA, USA) was performed on 4 µm thick sections of TMA slides after microwave antigen retrieval. For negative controls, tissue sections were incubated with antibody diluent (DAKO, Cambridge, UK) without primary antibody. Immunohistochemical scoring was performed by two pathologists (Jang KY and Kim KM) without the information of clinicopathological factors. To minimize inter-observer variability, two authors simultaneously counted PD1-positive cells and evaluated staining area and staining intensity for PD-L1 immunostaining with the consensus under the multi-viewing microscope (Nikon ECLIPSE 80i, Nikon, Tokyo, Japan). The consensus for immunostaining scoring was reached after discussion by two authors. To quantify tumor infiltrating PD1-positive lymphocytes, the number of PD1-positive lymphocytes were counted at the highest numbered five high-power fields (HPF) in each TMA core. Thereafter, the number of PD1-positive lymphocytes was scored by adding the number of PD1-positive lymphocytes of the two different TMA cores. The field diameter of HPF was 0.55 mm, and the area of one HPF was 0.238 mm^2^. Therefore, the total area analyzed per case for counting of PD1-positive lymphocytes was 2.376 mm^2^. For the evaluation of immunostaining for PD-L1, we scored staining intensity and the area of staining. The staining intensity scored as 0 (no staining), 1(weak staining), 2 (intermediate staining), and 3 (strong staining). The area of staining was scored as 0 (0-10% of the cells stained), 1 (11-33% of the cells stained), 2 (34-66% of the cells stained), and 3 (67-100% of the cells stained). Thereafter, the sum of intensity score and staining proportion score was used for further analysis. Subsequently, the immunohistochemical expression of PD-L1 was scored by adding the sum score of two different TMA cores. The maximum combined score was twelve and the minimum combined score was zero.

### Statistical analysis

The cut-off number for PD1-positive lymphocytes and the cut-off score for PD-L1 immunostaining were determined by the area under the curve (AUC) of the receiver operating characteristic (ROC) curve at the highest positive likelihood ratio point for overall survival (OS) (Figure S2). Because the ROC curve is a plot of the true positive rate (sensitivity) *versus* the false positive rate (1 - specificity) for the determination of the death of patients, the cut-off level for the ideal test is presented as the sensitivity 1 and specificity 1 (AUC 1.000). Therefore, we choose the cut-off point at the highest AUC value. The cut-off point of the PD1 immunostaining was one PD1-positive lymphocyte in 10 HPF from two TMA cores. The area under the curve was 0.719 for PD1 (Table S2). When there were one or more PD1-positive lymphocytes in two 3.0 mm cores, they were included in the PD1-positive subgroup. The cut-off point for the combined score of PD-L1 immunostaining was eight. The area under the curve was 0.736 for PD-L1 (Table S2). The immunostaining for PD-L1 was scored positive when the combined score was greater than or equal to eight. Pearson’s chi-square test was used to analyze the association between the immunohistochemical staining and variable clinicopathological factors. The end points of interest were OS and event-free survival (EFS). The follow-up end point was the date of last contact or death through March 2013. OS was measured from the date of diagnosis to the date of last follow-up or death by STS. Patients who were still alive at last contact or death from other causes were treated as censored for OS analysis. EFS was calculated as the time from diagnosis to the date of last follow-up, local relapse, late distant metastasis, or death from STS. Patients who were alive at last contact without local relapse or late distant metastasis or who died from other causes were treated as censored for EFS analysis. Univariate and multivariate Cox proportional hazard regression analyses were performed to estimate the impact on OS and EFS. Survival analysis also evaluated using Kaplan-Meier survival analysis. Statistical analysis performed by using SPSS software (version 18.0). *P* values less than 0.05 were considered statistically significant.

## Supporting Information

Figure S1 **Immunohistochemical expression of PD-L1 in intra-tumoral non-neoplastic cells.** A) In contrast to tumor cells, intra-tumoral non-neoplastic cells negative for PD-L1. B) In a PD-L1-negative case, tumor cells did not express PD-L1 but intra-tumoral endothelial cells are positive for PD-L1. C) Intra-tumoral endothelial cells (arrow) and inflammatory cells (arrow head) express PD-L1. Original magnification, x400.
(TIF)

Figure S2 **Analysis of sensitivity and specificity of PD1 and PD-L1 score for the event of overall survival (death of the patient) by receiver operator characteristic curves.** Arrow indicates a cut-off point for the number of intra-tumor PD1-positive lymphocytes and arrow head indicates a cut-off point for the PD-L1 immunostaining.
(TIF)

Table S1 **The association between histologic type of soft-tissue sarcoma and tumor stage.**
(DOC)

Table S2 **Statistical data on receiver operating characteristic curves.**
(DOC)
