# Supplementary figures and images for: Tumor Infiltrating PD1-Positive Lymphocytes and the Expression of PD-L1 Predict Poor Prognosis of Soft Tissue Sarcomas
Source: PLoS One. 2013 Dec 11;8(12):e82870. doi: 10.1371/journal.pone.0082870 (PMC3859621; doi:10.1371/journal.pone.0082870)

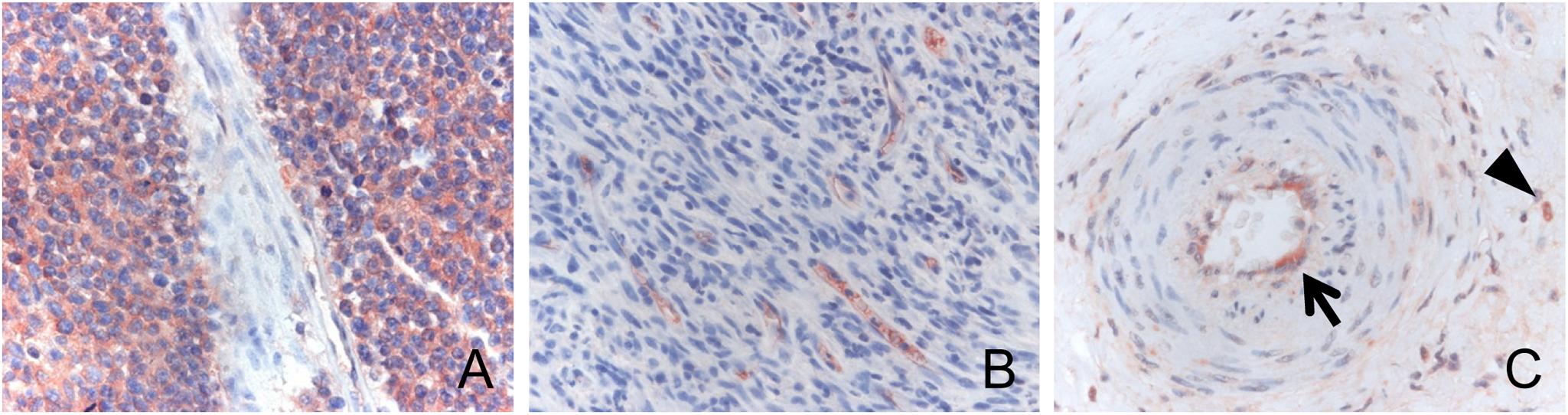

Supplement: Figure S1 — Immunohistochemical expression of PD-L1 in intra-tumoral non-neoplastic cells. A) In contrast to tumor cells, intra-tumoral non-neoplastic cells negative for PD-L1. B) In a PD-L1-negative case, tumor cells did not express PD-L1 but intra-tumoral endothelial cells are positive for PD-L1. C) Intra-tumoral endothelial cells (arrow) and inflammatory cells (arrow head) express PD-L1. Original magnification, x400. (TIF) [file pone.0082870.s001.tif]

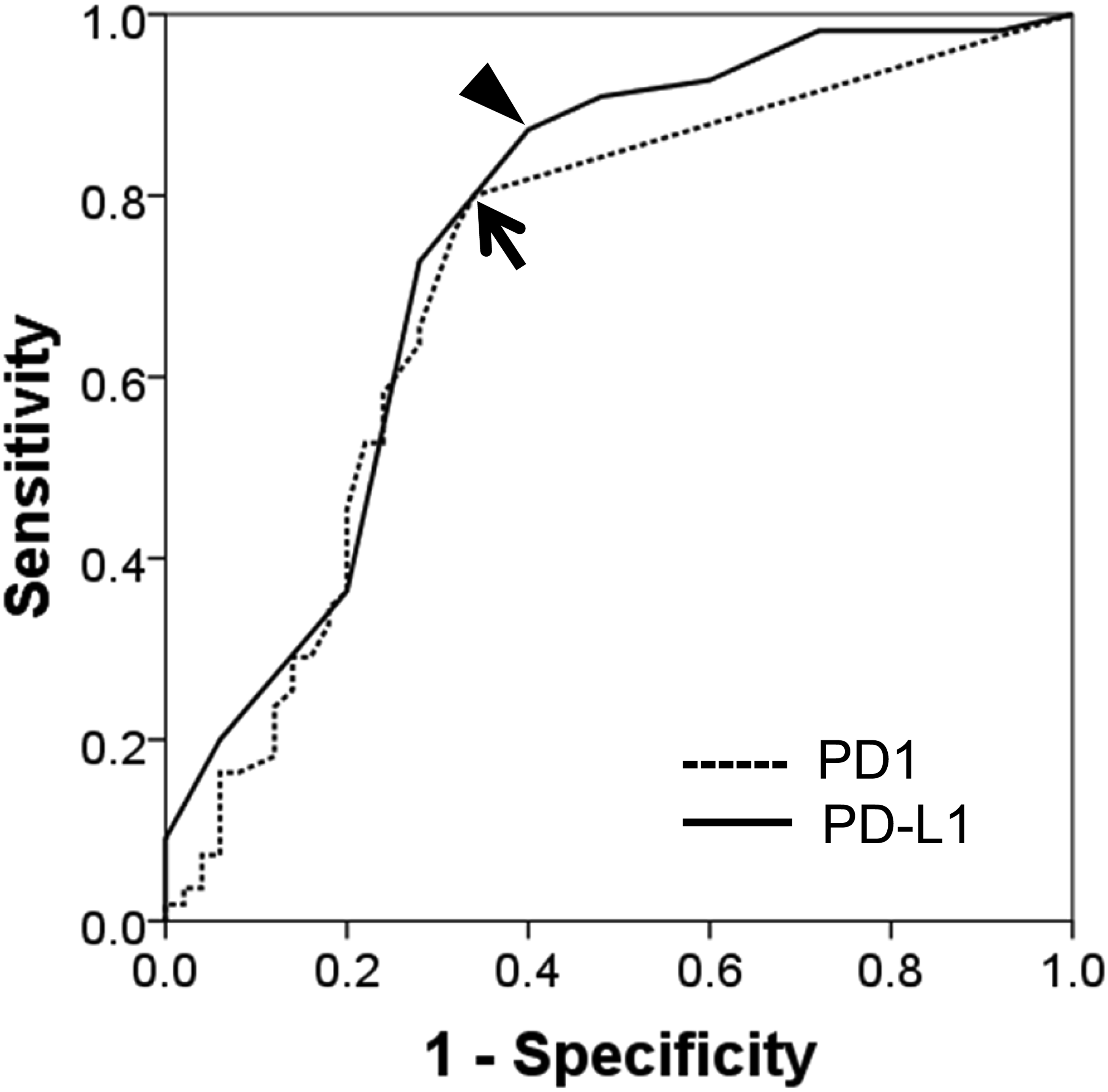

Supplement: Figure S2 — Analysis of sensitivity and specificity of PD1 and PD-L1 score for the event of overall survival (death of the patient) by receiver operator characteristic curves. Arrow indicates a cut-off point for the number of intra-tumor PD1-positive lymphocytes and arrow head indicates a cut-off point for the PD-L1 immunostaining. (TIF) [file pone.0082870.s002.tif]
