# Supplementary material for: Tumor Infiltrating PD1-Positive Lymphocytes and the Expression of PD-L1 Predict Poor Prognosis of Soft Tissue Sarcomas
Source: PLoS One. 2013 Dec 11;8(12):e82870. doi: 10.1371/journal.pone.0082870 (PMC3859621; doi:10.1371/journal.pone.0082870)
Supplement: Table S1 — The association between histologic type of soft-tissue sarcoma and tumor stage. (DOC) [file pone.0082870.s003.doc]

Table S1. The association between histologic type of soft-tissue sarcoma and tumor stage

| Histologic type | *N* | Stage | |
| --- | --- | --- | --- |
|  |  | Low (I/II) | High (III/IV) |
| Leiomyosarcoma | 20 | 4 (20%) | 16 (80%) |
| Synovial sarcoma | 16 | 10 (63%) | 6 (38%) |
| Undifferentiated sarcoma | 11 | 4 (36%) | 7 (64%) |
| Myxoid liposarcoma | 10 | 10 (100%) | 0 (0%) |
| Well differentiated liposarcoma | 4 | 3 (75%) | 1 (25%) |
| Dedifferentiated liposarcoma | 3 | 3 (100%) | 0 (0%) |
| Ewing sarcoma | 6 | 2 (33%) | 4 (67%) |
| Malignant peripheral nerve sheath tumor | 6 | 3 (50%) | 3 (50%) |
| Adult fibrosarcoma | 5 | 4 (80%) | 1 (20%) |
| Angiosarcoma | 5 | 1 (20%) | 4 (80%) |
| Myxofibrosarcoma | 4 | 4 (100%) | 0 (0%) |
| Epithelioid sarcoma | 4 | 1 (25%) | 3 (75%) |
| Alveolar rhabdomyosarcoma | 4 | 2 (50%) | 2 (50%) |
| Embryonal rhabdomyosarcoma | 2 | 0 (0%) | 2 (100%) |
| Pleomorphic rhabdomyosarcoma | 2 | 2 (100%) | 0 (0%) |
| Low grade myofibroblastic sarcoma | 2 | 1 (50%) | 1 (50%) |
| Clear cell sarcoma | 1 | 0 (0%) | 1 (100%) |
