# Supplementary material for: Tumor Infiltrating PD1-Positive Lymphocytes and the Expression of PD-L1 Predict Poor Prognosis of Soft Tissue Sarcomas
Source: PLoS One. 2013 Dec 11;8(12):e82870. doi: 10.1371/journal.pone.0082870 (PMC3859621; doi:10.1371/journal.pone.0082870)
Supplement: Table S2 — Statistical data on receiver operating characteristic curves. (DOC) [file pone.0082870.s004.doc]

Table S2. Statistical data on receiver operating characteristic curves

| Markers | Cut-off | AUC | *P* | 95% CI |
| --- | --- | --- | --- | --- |
| PD1 | ≥ 1/10 HPF | 0.719 | < 0.001 | 0.618-0.821 |
| PD-L1 | ≥ 7 | 0.715 | < 0.001 | 0.613-0.816 |
|  | ≥ 8 | 0.736 | < 0.001 | 0.638-0.835 |
|  | ≥ 9 | 0.724 | < 0.001 | 0.624-0.823 |

Abbreviations: AUC, area under the curve; 95% CI, 95% confidence interval.
